# Sociodemographic Factors and Stage of Cancer at Diagnosis: A Population-Based Study in South India

**DOI:** 10.1200/JGO.18.00160

**Published:** 2019-07-19

**Authors:** Aleyamma Mathew, Preethi Sara George, Kunnambath Ramadas, Beela Sarah Mathew, Aswin Kumar, Sivasevan Roshni, Krishnan Nair Lalithamma Jayakumar, Christopher M. Booth

**Affiliations:** ^1^ Regional Cancer Centre, Trivandrum, India; ^2^ Medical College, Trivandrum, India; ^3^ Queen’s University, Kingston, Ontario, Canada

## Abstract

**PURPOSE:**

Lower socioeconomic status is associated with inferior cancer survival in high-income countries, but whether this applies to low- and middle-income countries is not well described. Here, we use a population-based cancer registry to explore the association between educational level and stage of cancer at diagnosis in South India.

**METHODS:**

We used the Trivandrum District population-based cancer registry to identify all cases of breast and cervical cancer (women) and oral cavity (OC) and lung cancer (men) who were diagnosed from 2012 to 2014. Educational status—classified as illiterate/primary school, middle school, or secondary school or higher—was the primary exposure of interest. Primary outcome was the proportion of patients with advanced stage disease at diagnosis defined as stage III and IV (breast, cervix, or OC) or regional/metastatic (lung).

**RESULTS:**

The study population included 4,547 patients with breast (n = 2,283), cervix (n = 481), OC (n = 797), and lung (n = 986) cancer. Educational status was 22%, 19%, and 26% for illiterate/primary, middle, and secondary school or higher, respectively. Educational status was missing for 33% of patients. The proportion of all patients with advanced stage disease was 37% (breast), 39% (cervix), 67% (OC), and 88% (lung). Patients with illiterate/primary school educational status were considerably more likely to have advanced breast cancer (50% *v* 39% *v* 36%; *P* < .001), cervix cancer (46% *v* 43% *v* 24%; *P* = .002), and OC cancer (77% *v* 76% *v* 59%; *P* < .001) compared with patients with higher educational levels. The proportion of patients with advanced lung cancer did not vary across educational levels (89% *v* 84% *v* 88%; *P* = .350).

**CONCLUSION:**

A substantial proportion of patients in South India have advanced cancer at the time of diagnosis. This is particularly true among those with the lowest levels of education. Future health awareness and preventive interventions must target less-educated communities to reduce delays in seeking medical care for cancer.

## INTRODUCTION

Lower socioeconomic status (SES) is known to be associated with an increased incidence of cancer and inferior survival.^1-5^ Various hypotheses to explain survival differences between social groups have been proposed in the literature, including differences in tumor biology, patient comorbidity, stage of disease at diagnosis, access to therapy, and treatment practices.^2^

Difference in disease stage is a commonly cited potential mechanism for the observed relationship between SES and cancer outcomes.^2^ Whereas the association between SES, stage of cancer at diagnosis, and survival has been described in a number of studies from high-income countries (HICs), there is limited literature exploring these issues in low- and middle-income countries (LMICs). Thirty-nine studies were included in a 2006 systematic review of the socioeconomic inequalities in cancer survival—none of these studies came from LMICs.^2^ Given the explosive burden of cancer in LMICs; the unique health system challenges of LMICs; and the significant demographic, cultural, and socioeconomic differences between populations in HICs and LMICs, this area of investigation represents a critical unmet need. As LMICs develop cancer care health systems, it is critical to understand what subgroups within the general population might benefit most from educational and preventive interventions. We are not aware of any population-based study that has evaluated the association between SES, stage of cancer at diagnosis, and cancer survival in LMICs. In this report, we evaluate the association between sociodemographic factors and stage of cancer at diagnosis in Trivandrum District in South India. We chose to focus this study on breast and cervical cancer in women and lung and oral cavity cancer in men, as they represent the highest burden of cancer in India.

## METHODS

### Study Population

The Population-Based Cancer Registry (PBCR) of Trivandrum District in South India, located in the Regional Cancer Centre (RCC), Trivandrum, was used to identify all incident cases of breast and cervical cancer in women and oral cavity and lung cancer in men who were diagnosed from 2012 to 2014. Trivandrum District has a population of 3.3 million—54% urban and 46% rural—and is located in the southern Indian state of Kerala (population 33 million). Kerala has the highest literacy rate (94% *v* 73% national rate), greatest life expectancy (age 74 years *v* 64 years), and lowest infant mortality rate in India.^6^

### Data Sources

The Trivandrum District PBCR is one of 27 cancer registries operating under the National Cancer Registry Program of India. The PBCR uses an active case-finding methodology that consists of visiting government/private hospitals and pathology laboratories. Data have been collected from more than 60 hospitals and seven pathology laboratories. Computerized information processing includes linkage of patient data obtained from various sources and review of duplicate/redundant records. Validity of the data is monitored by conducting data quality exercises periodically on abstraction of data from medical records and coding of the diagnosis. Microscopic confirmation, death certificate only, fatality ratio (%), and the proportion of unknown primary sites are used to assess the quality of the registry.

Major sources for incidence data are the RCC (63% of cases) and the Government Medical College Hospital (24% of cases), both of which are located in Trivandrum. A large number of private hospitals (n = 47) and government hospitals (n = 32) also diagnose and treat patients with cancer. As cancer is not a notifiable disease in India, registration of incident cancer cases is carried out using active case finding. On the basis of an administrative letter provided by the Principal Secretary, Government of Kerala, to all health authorities in the district, cooperation from all hospitals has been obtained. The Trivandrum District PBCR employs 14 tumor registrars who are trained in cancer registration in locally and nationally organized courses, followed by continuing in-service training. PBCR staff review medical records from 60 potential data sources and seven pathology laboratories at regular intervals to abstract data on incident cancer cases. Information collected includes age, residential address, gender, religion, marital status, education, date of incidence, basis of diagnosis, topography, morphology, clinical extent of disease, treatment details, and vital status.

### Definitions of Exposures and Outcomes

Sociodemographic characteristics are captured routinely by PBCR staff. In this study, the association between stage of disease and the following sociodemographic characteristics were considered: age, marital status, religion, rural/urban residence, and education. Self-reported educational levels include illiterate, up to primary school, up to middle school, up to secondary school, and college and technical school. Patients were classified into three groups: illiterate/primary (0 to 5 years), middle school (6 to 10 years), or secondary school or greater (> 10 years). Information regarding household income was not considered in this study as a result of high rates of missing data and concerns regarding the validity of self-reported income.

Stage at diagnosis is routinely assigned by the treating oncologist for all patients seen at RCC and Government Medical College Hospital. On the basis of review of the case sheet, registry staff record clinical stage at time of diagnosis. If clinical stage is not available, pathologic stage is recorded. Staging systems vary by disease site—breast and oral cavity cancer are staged using the TNM classification system, cervical cancer is staged using the International Federation of Gynecology and Obstetrics system, and lung cancer is staged using the SEER clinical extent of disease system.

In cases of missing data, PBCR staff reviewed primary data sources to assign stage of disease and contacted patients by telephone to ascertain educational status. Among a random sample of 10% of patients, the agreement rate between oncologist-assigned stage and PBCR stage grouping was high: 92% for breast, 91% for cervix, 89% for oral cavity, and 88% for lung cancer. The primary end point of the study was to evaluate the association between stage of cancer at diagnosis and educational level. We also considered the following sociodemographic factors: age, marital status, religion, and urban/rural residence.

### Statistical Analysis

We compared proportions between sociodemographic groups using the χ^2^ test. We used the Marascuilo procedure to compare multiple proportions.

*P* values < .05 were deemed statistically significant. No adjustments were made for multiple comparisons.

## RESULTS

### Characteristics of Study Population

The study population included 4,547 patients with breast (n = 2,283), cervix (n = 481), lung (n = 986), and oral cavity (n = 797) cancer who were diagnosed in Trivandrum District from 2012 to 2014. Characteristics of the study population are listed in Table 1. The mean age was 58 years and 31% of cases were age 65 years or older. Eighty-three percent of patients were married. The majority (74%) of patients are Hindu and 26% of patients attended secondary school. Within the four cancer subgroups, patients with breast cancer were younger, more likely to live in urban areas, and more highly educated. A lower proportion of women with cervical cancer were married relative to those with the other cancers. Sixty-three percent (2,868 of 4,547) of patients were identified from RCC, 24% (1,090 of 4,547) from Government Medical College Hospital, and 13% (589 of 4,547) from other sources. Rates of data capture for stage and educational level were high for cases seen at RCC (range, 96% to 99% and 99% to 100%, respectively). Among patients seen at Government Medical College Hospital, the stage and educational capture rates were 62% to 92% and 7% to 12%, respectively, and corresponding rates from other sources were 35% to 71% and 12% to 19%.

**TABLE 1:**
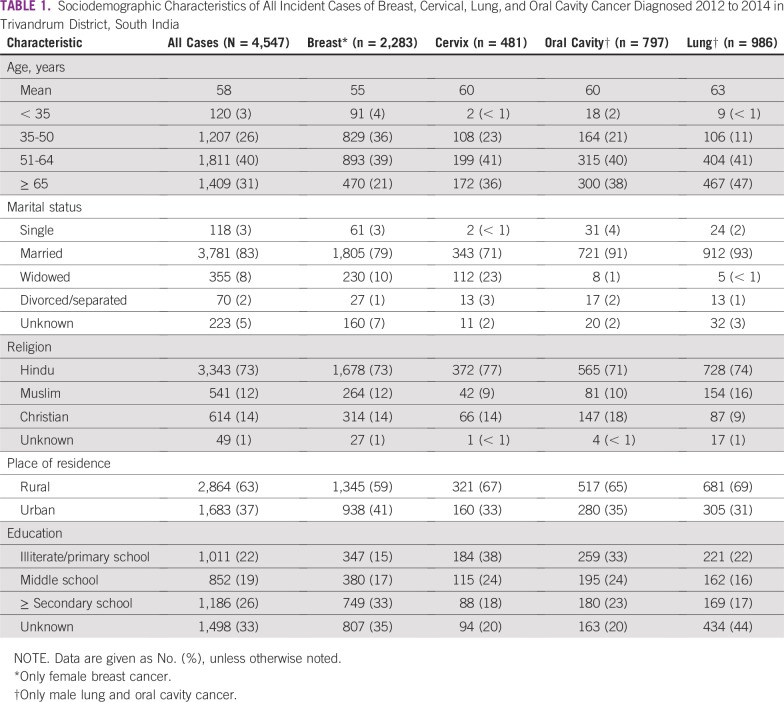
Sociodemographic Characteristics of All Incident Cases of Breast, Cervical, Lung, and Oral Cavity Cancer Diagnosed 2012 to 2014 in Trivandrum District, South India

### Stage of Cancer at Diagnosis

As listed in Table 2, only 8% to 14% of patients presented with early-stage disease—that is, stage I/localized disease. Whereas 10% and 13% of patients with breast and cervical cancer presented with stage IV disease, the rate of metastatic disease at diagnosis was much higher for those with oral cavity (40%) and lung (46%) cancers. Overall, the proportion of patients with advanced disease—that is, stage III and IV or regional/metastatic—was 37% for breast, 39% cervix, 67% oral cavity, and 88% lung cancer.

**TABLE 2:**
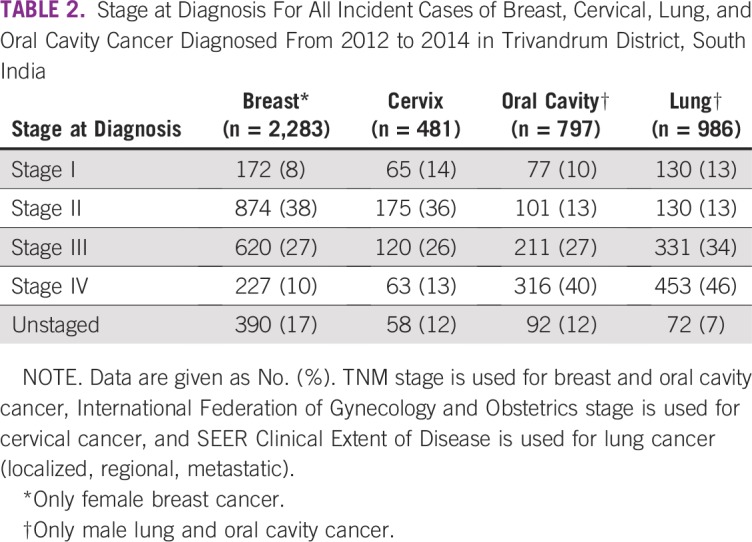
Stage at Diagnosis For All Incident Cases of Breast, Cervical, Lung, and Oral Cavity Cancer Diagnosed From 2012 to 2014 in Trivandrum District, South India

### Sociodemographic Factors and Stage of Cancer

**FIG 1:**
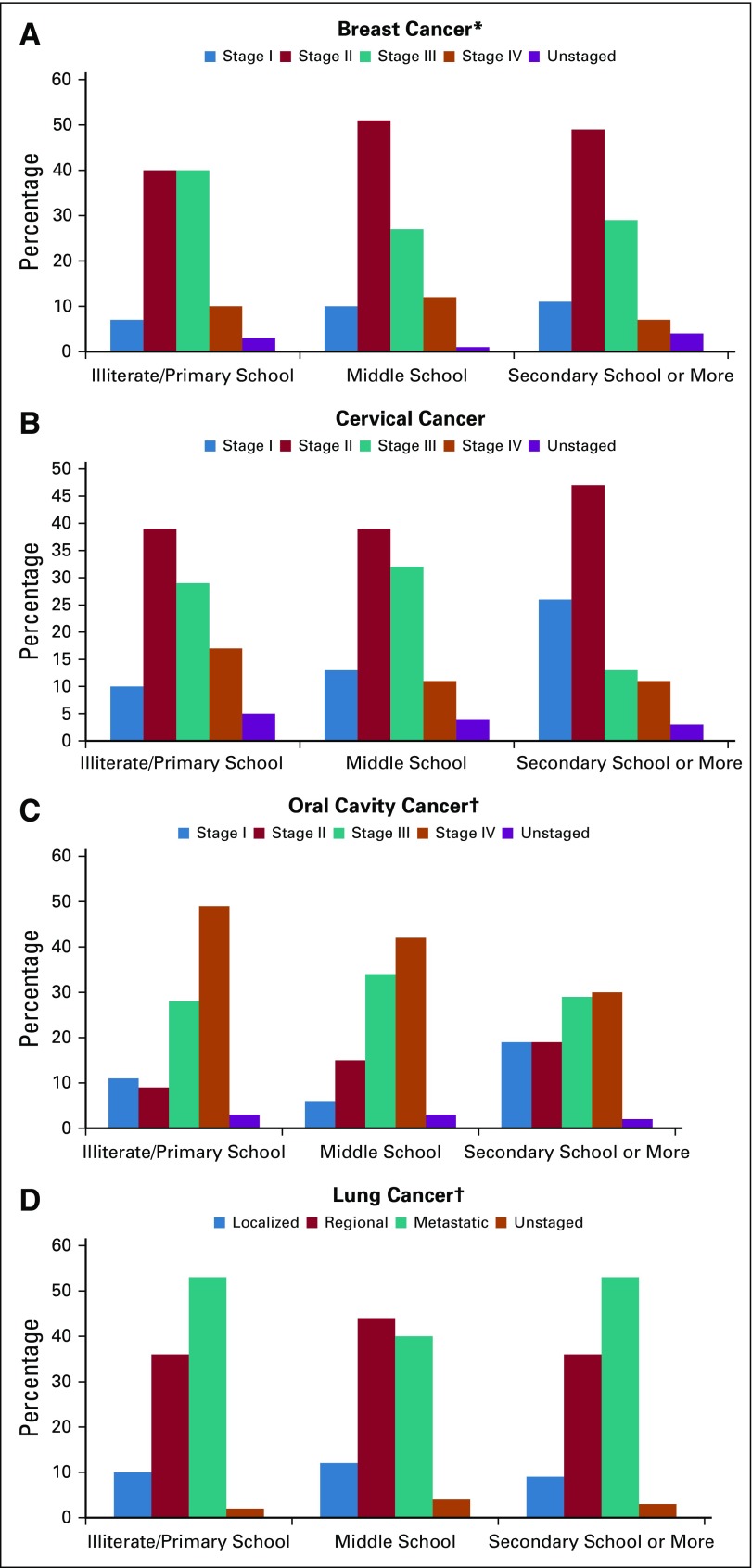
Distribution of stage at diagnosis by educational level among all incident cases of breast, cervical, lung, and oral cavity cancer diagnosed from 2012 to 2014 in Trivandrum District, South India. (A) Breast cancer. (B) Cervical cancer. (C) Oral cavity cancer. (D) Lung cancer. (*) Only female breast cancer. (†) Only male lung and oral cavity cancer.

Distribution of stage at diagnosis by educational level is shown in Table 3 and Figure 1. The proportion of patients with advanced disease—stage III and IV or regional/metastatic—is shown in Table 4. For breast, cervix, and oral cavity cancer, there is substantial association between stage at diagnosis and educational level. Among patients with breast cancer, the proportion of patients with advanced disease was 50%, 39%, 36% (*P* < .001) for illiterate/primary school, middle school, and secondary school or greater, respectively. Corresponding figures for cervix and oral cavity cancer are 46%, 43%, and 24% (*P* = .002) and 77%, 76%, and 59% (*P* < .001), respectively. The proportion of patients with lung cancer with advanced disease did not differ substantially across educational groups (89%, 84%, and 88%, respectively; *P* = .350).

**TABLE 3:**
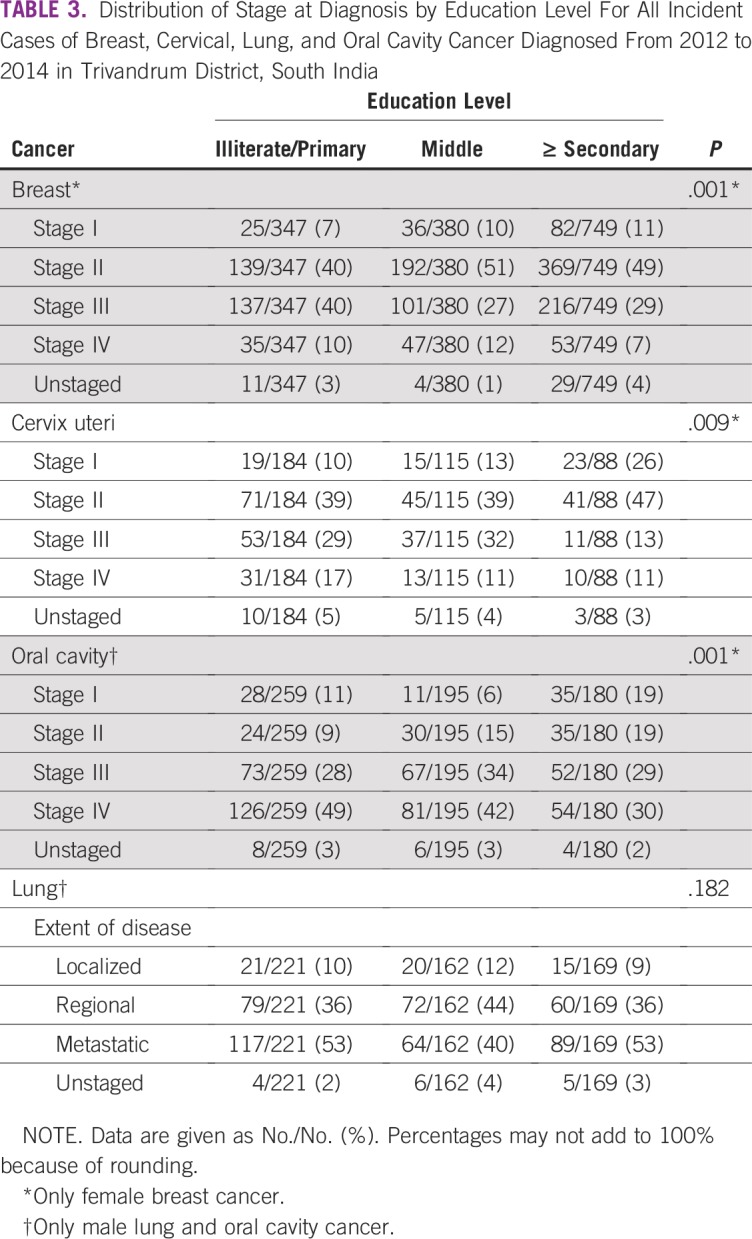
Distribution of Stage at Diagnosis by Education Level For All Incident Cases of Breast, Cervical, Lung, and Oral Cavity Cancer Diagnosed From 2012 to 2014 in Trivandrum District, South India

**TABLE 4:**
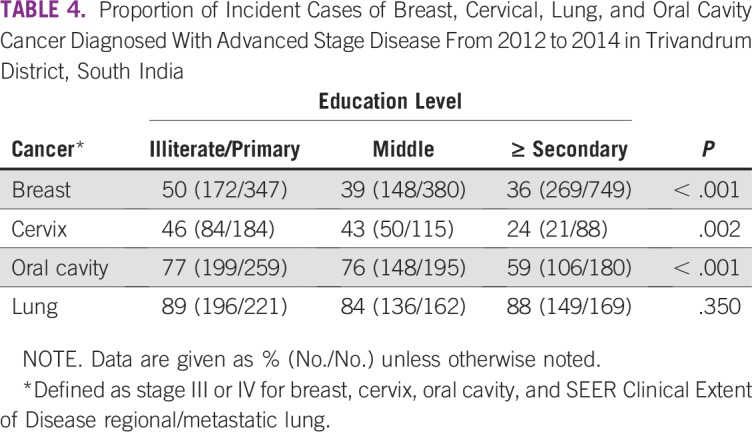
Proportion of Incident Cases of Breast, Cervical, Lung, and Oral Cavity Cancer Diagnosed With Advanced Stage Disease From 2012 to 2014 in Trivandrum District, South India

Association between advanced stage of disease and age, marital status, religion, and rural/urban residence is shown in Table 5. Older men (age > 50 years) with oral cavity cancer were more likely than younger patients to have advanced disease (52% *v* 42%; *P* = .035).

**TABLE 5:**
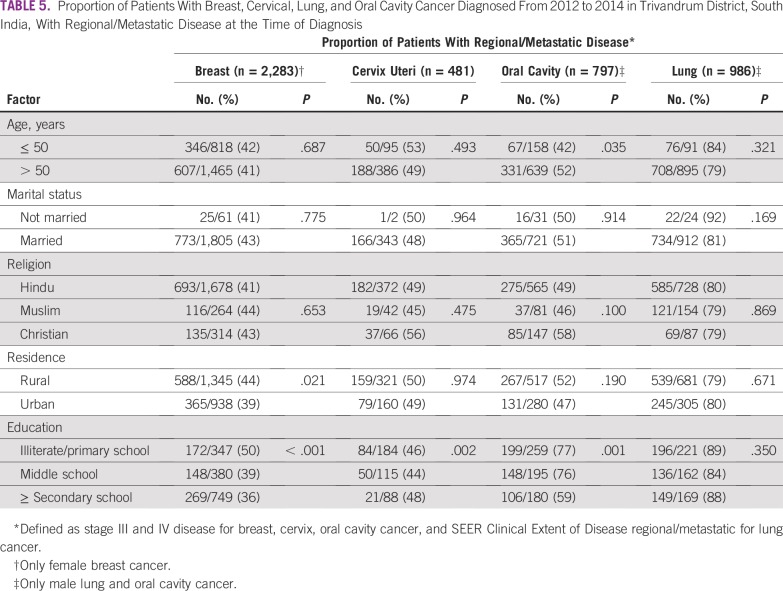
Proportion of Patients With Breast, Cervical, Lung, and Oral Cavity Cancer Diagnosed From 2012 to 2014 in Trivandrum District, South India, With Regional/Metastatic Disease at the Time of Diagnosis

## DISCUSSION

In this study, we used data from a PBCR in South India to evaluate whether there was an association between educational status and stage of cancer at diagnosis. Our study focused on the four most common cancers in India. Several important findings have emerged. First, our results demonstrate that the proportion of patients who are diagnosed with advanced stage disease in the general population of India is higher than in HICs. Second, our study demonstrates a strong association between educational status and stage of breast, cervical, and oral cavity cancer at diagnosis. This has important policy and public health implications. Finally, we did not observe any such association for patients with lung cancer.

It is worth comparing the distribution of stage at diagnosis in our study with existing literature. Eight percent of women in our study with breast cancer had stage I disease and 37% had stage III and IV disease, which is consistent with other reports from India^7^; however, these figures are in stark contrast to data from HICs, where 30% to 45% of women have stage I disease and 8% to 22% stage III and IV disease at diagnosis.^8^ Likewise, we report that 14% and 39% of women with cervical cancer had stage I and stage III and IV disease, respectively—these data are consistent with that another study from India.^7^ However, corresponding figures in HICs are different; in Canada, 34% of women have stage I disease and 19% of women have stage III and IV disease at diagnosis.^9^ These differences in stage of cancer at diagnosis between India and HICs likely reflect differences in cancer awareness/education, availability of screening programs, and access to cancer care.

Stage distributions of oral cavity cancer and lung cancer in our study are less different than those reported in HICs. In our cohort, 10% and 67% of men with oral cavity were diagnosed with stage I or stage III and IV cancer, respectively. Whereas the proportion of patients with stage I disease in our study is lower than that in the United States (10% *v* 29%), the proportion of patients with stage III and IV disease is similar (67% *v* 67%).^10^ Distribution of stage at diagnosis for lung cancer observed in our cohort—13% localized and 80% regional/metastatic—is also similar to reports from Canada and the United States, where 65% and 66% of patients have stage III and IV disease, respectively.^11,12^

The primary objective of our study was to evaluate the extent to which stage of cancer at diagnosis was associated with educational status, which was chosen as a surrogate for SES. Parallel work in HICs commonly use neighborhood median household income from census data as a measure for SES.^5,13,14^ Comparable census data in South India were lacking and we had concerns regarding the validity of self-reported household income in the Indian context. For these reasons, we elected to use educational level, commonly used in epidemiologic studies as a surrogate for SES.^15^ Public health studies in India have demonstrated that educational level in India is correlated with other measures of SES, including occupation, housing, and social status.^16^ Educational level is a strong determinant of future employment and income. Education is also associated with cognitive function and health literacy, which can affect health-seeking behaviors.^17^

Our study results demonstrate a substantial association between low educational status and advanced stage cancer at diagnosis for patients with breast, cervical, and oral cavity cancers. This association was not observed in patients with lung cancer. These results have face validity when one considers the mechanism by which educational status may be related to stage of disease. Patients with greater education who develop early signs of breast cancer (ie a breast lump), cervical cancer (ie, abnormal vaginal bleeding), and oral cancer (ie, a mouth lesion) may have greater awareness and access to health care, thus seeking earlier medical treatment and receiving a diagnosis with earlier disease than patients from lower educational backgrounds. However, for patients with lung cancer, by the time symptoms manifest, the disease is often already advanced, in which case early treatment-seeking behavior would be expected to have less effect on stage at diagnosis.

In their overview of the existing literature, Woods et al^2^ conclude that stage of disease at diagnosis and access to optimal treatment explain a portion of the disparity in the survival of patients with cancer. Whereas much of the literature from the United States has found an association between SES and stage of disease at diagnosis,^2,13,18^ several large studies conducted in the United Kingdom and Canada have failed to confirm this observation.^5,14,19,20^ It remains unknown whether the results in Canada and the United Kingdom are different from the findings in the United States because of the availability of universal health insurance in the former but not the latter. We are not aware of any studies in LMICs exploring the association between stage, SES, and survival. Results presented in this study represent the first step in doing so. Future work should explore survival differences across socioeconomic groups and the relative extent to which stage and treatment delivery explain any observed survival differences.

Several hospital-based studies in India have explored the association between educational level and stage of cancer at diagnosis. Sathwara et al^21^ evaluated the association between sociodemographic factors and late-stage disease among 1,210 women with breast cancer who were treated at Tata Memorial Hospital (TMH) in 2008. Fifty-four percent of patients had advanced—that is, stage III and IV—disease, and rural residence and illiterate educational status were independently associated with advanced disease at diagnosis. In a similar study, Jain et al^22^ describe factors associated with advanced cervical cancer at diagnosis among 765 women who were treated at TMH in 2007. Fifty-two percent of all women had stage III and IV disease at diagnosis. This rate was higher among illiterate women compared with women with secondary education (59% *v* 44%; *P* = .010). Of note, overall rates of stage III and IV breast and cervical cancer in these institution-based series from TMH (54% and 52%) are considerably higher than those reported in our study (37% and 39%), which may themselves reflect regional differences in SES between the Indian states of Maharashtra and Kerala.

Two studies have used the Hospital-Based Cancer Registry at the RCC in Trivandrum, Kerala, to explore this issue. Among 522 patients with breast cancer seen at RCC in 2006, Ali et al^23^ found that advanced disease stage was more common among women who were not married and who had lower educational levels. Kaku et al^24^ reported similar findings in their study of 349 women with cervical cancer seen at RCC in 2006. In their systematic review of barriers to breast cancer care in LMICs, Sharma et al^25^ identified eight studies (sample size range, 66 to 903) that demonstrated an association between SES and delayed presentation of breast cancer. The authors concluded that there was strong evidence that lower income and education contributed to barriers in breast cancer care. The existing literature is limited by the fact that, with one exception,^26^ each of the studies in the meta-analysis were institution based and therefore potentially limited by referral bias and selection bias. Given that lower SES is likely a major barrier to accessing cancer health services in LMICs, a population-based study, rather than a hospital-based study, is the optimal design. This is a notable strength of our current study.

Our study does have methodological limitations that warrant comment. As shown in Appendix Table A1, stage and educational level were more likely to be missing among those patients who were treated at the Government Medical College Hospital or other smaller institutions compared with the RCC in Trivandrum. This may limit the generalizability of our study results. Moreover, our study cannot explain why patients with lower educational status are more likely to have advanced cancer. It is likely that this reflects a delay in seeking medical attention, but future qualitative work is needed to explore this more fully to use this knowledge to improve the care of patients from impoverished backgrounds.

Cancer has emerged as a major cause of morbidity and mortality in LMICs where patients are more likely to be diagnosed with advanced cancer than in HICs. Optimal development and implementation of cancer control systems requires an understanding of existing patterns of disease, treatment, and outcomes in LMICs. Health services research can make use of existing data in cancer registries to provide unique insights into health system performance in LMICs.^27-29^ Whereas the association between SES, stage of cancer, and survival has been well described in HICs, there are limited reports from LMICs. Data from this study suggest that future health awareness, preventive, and treatment interventions in Kerala will need to specifically target less-educated communities in an effort to minimize delays in seeking medical care for cancer.

## Figures and Tables

**TABLE A1:**
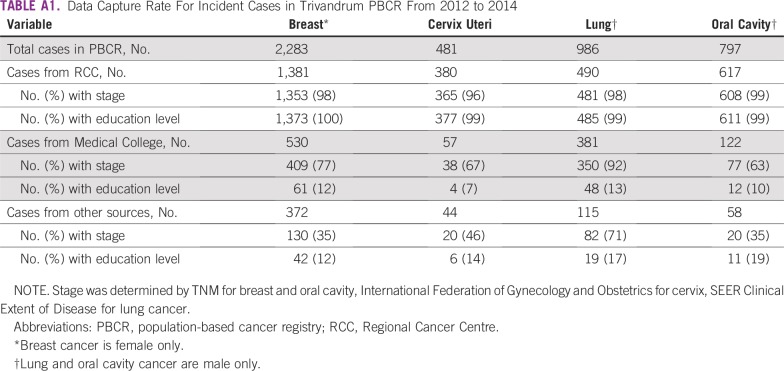
Data Capture Rate For Incident Cases in Trivandrum PBCR From 2012 to 2014
